# Prediction of body condition score throughout lactation by random regression test‐day models

**DOI:** 10.1111/jbg.12890

**Published:** 2024-08-31

**Authors:** H. Atashi, Y. Chen, J. Chelotti, P. Lemal, N. Gengler

**Affiliations:** ^1^ TERRA Teaching and Research Center, Gembloux Agro‐Bio Tech University of Liège Gembloux Belgium; ^2^ Department of Animal Science Shiraz University Shiraz Iran; ^3^ Instituto de Investigación en Señales Sistemas e Inteligencia Computacional, sinc(i), FICH‐UNL/CONICET Santa Fe Argentina

**Keywords:** Holstein, management, random regression, test‐day yield

## Abstract

Regular monitoring of body condition score (BCS) changes during lactation is a crucial management tool in dairy cattle; however, the current BCS measurements are often discontinuous and unevenly spaced in time. The aim of this study was to investigate the ability of random regression test‐day models (RR‐TDM) to predict BCS for the entire lactation in dairy cows even if the actual scoring is limited to one BCS record. The data consisted of test‐day records of milk yield (MY), fat percentage (FP), protein percentage (PP) and BCS (based on a 9‐point scale with unit increments; 1–9) collected from 2014 to 2022 in 128 herds in the Walloon Region of Belgium. In total, 20,698 test‐day records on 2166 first‐parity Holstein cows (2–12 with an average of 9.42 test‐day records per cow) were available for MY, FP and PP; and 7985 records on the same animals (2–12 with an average of 3.68 records per cow) were available for BCS. To estimate the solutions, only one randomly selected BCS record per animal along with all her MY, FP and PP records were used, which were then used to predict BCS data (calibration set). The remaining BCS (1–11 with an average 2.86 BCS records per animal) were used to evaluate the goodness of the predictions (validation set). Multiple‐trait RR‐TDM was used to estimate (co)variance components through the average information restricted maximum likelihood (AI‐REML) algorithm. Predicted BCS were grouped into nine classes as the original observed BCS used for comparison. Pearson correlation between the predicted and observed BCS, prediction error (PE), absolute prediction error (APE) and root mean squared prediction error (RMSE) were calculated. Mean (standard deviation; SD) BCS was 4.97 (1.01), 4.95 (1.07) and 4.98 (1.00) BCS units in the full, calibration and validation datasets, respectively. Pearson correlation between the observed and predicted BCS was 0.71, mean (SD) PE was 0.04 (0.52) BCS units, mean (SD) APE was 0.48 (0.53) BCS units and RMSE was 0.72 BCS units. These findings demonstrate the ability of RR‐TDM to predict BCS for the entire lactation using a single BCS record along with available test‐day records of milk yield and composition in Holstein dairy cows.

## 1 INTRODUCTION

The body condition score (BCS) is a subjective metric routinely used worldwide to assess the body reserves of individual cows. Using formulated rations, appropriate feeding systems and good management allow cows to maintain at their ideal body condition throughout the lactation cycle. BCS reflects the body reserves in cows and is a good predictor of health and welfare and is recognized by animal scientists and producers as the most useful management tool in dairy cattle management (Dechow et al., 2004). The associations between BCS and milk production, postpartum anestrous, the risk of uterine infection and the risk of metabolic disorders have been documented (Berry et al., 2007; Roche et al., 2013; Souissi & Bouraoui, 2019; Waltner et al., 1993), justifying the interest in BCS as a trait for genetic evaluations. Furthermore, monitoring individual cow body fat and maintaining adequate body condition is essential to maintain a productive animal that has appropriate nutrition and fertility, while also producing acceptable amounts of milk (Berry et al., 2003; Roche et al., 2007; Zink et al., 2011). Thus, BCS is considered as an important candidate to be incorporated into decision support systems in the near future to help producers in making decisions. It is important to emphasize that a BCS can provide a historical view of what has happened with the animal in recent weeks and tracking changes in BCS is probably of greater value than identifying absolute measures of body condition (Bewley & Schutz, 2008; Garnsworthy, 2006). BCS may also be a valid indicator of animal welfare and indirectly of environmental factors influencing welfare (e.g. feeding and heat stress); however, further research is needed to determine the effect of BCS and BCS change on animal welfare (Mee & Boyle, 2020). Therefore, having continuously available BCS measurements would be a major interest for dairy herd management. Body condition scoring is traditionally assessed by either visual appraisal or by feeling the spinous processes of the loin and around the tail area; and despite being subjective, BCS is currently a favoured practical method of evaluating the proportion of body fat.

In most countries, cows are given a BCS based on a 5‐point scoring system (1–5) with quarter points (Edmonson et al., 1989; Wildman et al., 1982); however, there are various BCS systems evolved across the world such as a 6‐point scale (0–5) proposed in the United Kingdom (Mulvany, 1977), an 8‐point scale (1–8) developed in Australia (Earle, 1976) and a 10‐point scale (1–10) introduced in New Zealand (Macdonald & Roche, 2004). Dairy cows are given a BCS based on a 9‐point scale with unit increments (1–9) in the Walloon Region of Belgium (Bastin et al., 2007). Regardless of the scale used to measure BCS, low values always reflect emaciation and high values equate to obesity (Bastin et al., 2007; Roche et al., 2009; Wildman et al., 1982). Although the traditional body condition scoring is an easy‐to‐learn technique, this approach requires labour time and thus the BCS data recorded by producers are lacking for inclusion in genetic evaluations or into decision support systems. The potential ability of various methods such as utilizing digital image and machine learning algorithms, as well as using live body weight, milk yield and composition and mid‐infrared (MIR) spectrometry data for predicting BCS has been considered (De Vries et al., 2013; Frizzarin et al., 2023; Martins et al., 2020). However, these procedures need an initial capital cost as well as routine maintenance costs. Therefore, animal scientists are looking for a solution which can predict accurate BCS data routinely at little to no marginal cost.

Although test‐day model (TDM) is an effective tool for genetic evaluation in dairy cattle, its application for herd management purposes has not been emphasized. TDM could allow the prediction of future yields or the extension of incomplete lactation records; however, it needs a simple reparameterization as described by Mayeres et al. (2002). The contemporary group is used to remove biases from genetic evaluations; however, its definition in TDM is often problematic as it can be defined as either fixed or random. Considering contemporary groups as fixed removes bias caused by the association between effects corresponding to contemporary groups and sires. Considering contemporary groups as fixed removes bias caused by the association between effects corresponding to contemporary groups and sires and it has the advantage that expected breeding value is not a function of fixed effects (Van Vleck, 1987). However, the use of fixed effects may cause problems when dealing with small classes, as would be the case with small herds. On the other hand, considering contemporary groups as random could result in an increased effective number of daughters but at the expense of potential bias (Van Vleck, 1987). In test‐day model (TDM), fixed herd‐test‐day (HTD) effect has been widely used since the early days of test‐day modelling (Ptak & Schaeffer, 1993). This effect theoretically allows unbiased comparison of animals, but, for some cases, especially those with small herd size, the inclusion of the HTD effect as the contemporary group may not be an optimal choice (Swalve, 1995). Mayeres et al. (2002) proposed a straightforward modification by substituting the HTD fixed effect with three herd‐test‐related effects: a fixed herd‐test‐month period (HTMp) effect, a fixed herd‐test‐year (HTY) effect and a random herd‐test‐day (HTDr) effect. The HTMp and HTY effects consider the herd level and its potential seasonal trend, while HTDr effect considers the effect of the herd at a specific test date, which is not assigned to HTM and HTY effects. By considering HTDr as random, the solutions for the HTDr effect are regressed towards zero for the HTD classes.

The aim of this study was to investigate the ability of random regression test‐day models (RR‐TDM) to predict BCS for the entire lactation in Holstein cows based on one randomly selected BCS record and their routinely recording information.

## 2 MATERIALS AND METHODS

### 2.1 Phenotypic data

The data used consisted of test‐day records of milk yield (MY), fat percentage (FP), protein percentage (PP) and BCS collected from 2014 to 2022 on first‐parity Holstein cows distributed in 128 herds in the Walloon Region of Belgium. Age at the first calving was calculated as the difference between calving date and birth date and restricted to the range of 540–1200 days. Daily MY, FP and PP were restricted to range from 3 to 70 kg, 1%–9% and 1%–7%, respectively. The dataset was edited to accommodate records between 5 and 365 days in milk (DIM). Cows were required to have records for MY, FP, PP and BCS for at least two test days. Herds with fewer than 10 cows were removed from the data set.

The final dataset comprised 20,696 test‐day records on 2166 dairy cows (2–12 with an average of 9.56 test‐day records per cow) for MY, FP and PP; and 7985 records (2–12 with an average of 3.68 records per cow) for BCS. The exact DIM of the milk traits and BCS records varied from cow to cow. All MY, FP and PP records available for the included animals were used for variance component estimations. However, only one BCS record per animal was randomly selected and used for the estimation of the variance components and the solutions, which were then used to predict the remaining data. The remaining BCS records (1–11 with an average of 2.68 records per animal) were used to evaluate the goodness of the predictions (Hereafter, we call them ‘**ObsBCS**’). Pedigree depth of the animals was traced back as far as available to include all ancestors of the animals. Full pedigree records included 20,543 animals (4384 males).

### 2.2 Variance component estimation

In this study, the (co)variance components were estimated based on a multiple‐trait random regression test‐day model (RR‐TDM) using the second‐order polynomials (Mayeres et al., 2002) and the remodelled HTD effect suggested by Mayeres et al. (2002). The matrix notation of the model is:

$$y=\mathrm{Xb}+\mathrm{Ut}+Q\left(W_{1}h+W_{2}p+\mathrm{Za}\right)+e$$

where **y** is the vector of observations, **b = [μ, HTY, HTMp, AS, LS]** ^**t**^ is the vector with fixed effects, where **μ** = the overall mean, **HTY** = herd‐test‐year‐period (two classes were defined for test‐year‐period: test years of 2014–2016 and 2017–2022); **HTMp** = herd‐test‐month‐period (defined as the herd‐test‐season: winter from January to March, spring from April to June, summer from July to September and autumn from October to December); **AS** = calving‐age‐calving‐season (defined as age at calving class (three classes were created for age at calving) × season of calving × major lactation stage (three classes: DIM 5–50, 51–200 and 201–365)); **LS** = minor lactation stage to model the average lactation curve (DIM 5–15, 29 10‐day classes for DIM 16–305 and two 30‐day classes for DIM 306–365); **t** is the vector of the random herd‐test‐day effect (HTDr); **h** is the vector of common herd‐calving‐year‐period (HY) environmental random regression coefficients (three classes were defined for HY: calving years of 2014, 2015–2017 and 2018–2022); **p** is the vector of permanent environmental random regression coefficients; **a** is the vector of genetic random regression coefficients; **e** is the vector of residual effects; **X**, **U W**_**1**_, **W**_**2**_ and **Z** are the corresponding incidence matrices; and **Q** is the covariate matrix for second‐order Legendre polynomials. Covariances across all these random and the residual effects and the four traits were represented symbolically as follows:

$$\mathrm{Var}\left[\begin{array}{c} t\\ \mathrm{hy}\\ p\\ a\\ e \end{array}\right]=\left[\begin{array}{ccccc} D⊗I & 0 & 0 & 0 & 0\\ 0 & \mathrm{HY}⊗I & 0 & 0 & 0\\ 0 & 0 & P⊗I & 0 & 0\\ 0 & 0 & 0 & G⊗A & 0\\ 0 & 0 & 0 & 0 & R \end{array}\right]$$

Where, expressing the covariances between the four traits, **D** is the 4 × 4 covariance matrix among herd‐test‐day effects, **HY** is the 12 × 12 covariance matrix among herd‐calving‐year‐period regression coefficients, **P** is the 12 × 12 covariance matrix among permanent environmental regression coefficients, **G** is the 12 × 12 covariance matrix among additive genetic regression coefficients; **A** is the numerator relationship matrix based on the pedigree, ⊗ represents the Kronecker product function, **I** are identity matrices representing the number of levels for each effect and the number of observation for the residuals, and **R** contains residual covariances between traits (4 × 4 (co)variance matrix). Based on the initial test and to decrease model complexity, the residual variance for each trait was assumed to be homogeneous throughout lactation. Variance components were estimated using the average information REML (AIREML) algorithm implemented in the blupf90 family programs (Misztal et al., 2018).

### 2.3 Computation and validation of BCS predictions

The same multi‐trait linear model was used to predict BCS test‐day records as described by Mayeres et al. (2002). Estimates of all effects resulted directly from the solutions of the model used for estimation of variance components. This procedure theoretically allowed the prediction of BCS records for all possible DIM as all fixed effects were defined independently from a specific test‐date. However, because only monthly milk recordings were available, BCS predictions were restricted to the specific herd‐test dates aligned with milk yield and composition records. Vector of BCS estimates (${\hat{y}}_{\mathrm{BCS}}^{*}$), hereafter called ‘**prdBCS’**, was generated using the following formula:

$${\hat{y}}_{\mathrm{BCS}}^{*}=X^{*}{\hat{b}}_{\mathrm{BCS}}+U^{*}{\hat{t}}_{\mathrm{BCS}}+{W_{1}}^{*}{\hat{h}}_{\mathrm{BCS}}+{W_{2}}^{*}{\hat{p}}_{\mathrm{BCS}}+Z^{*}{\hat{a}}_{\mathrm{BCS}}+{\hat{e}}_{\mathrm{BCS}}^{*}$$

where all incidence matrices marked by the a ‘*’ related missing observations of BCS to relative solutions. Then, the predicted BCS values were rounded off to the nearest integer. Estimates of residuals $\left({\hat{e}}_{\mathrm{BCS}}^{*}\right)$ for BCS at these specific herd‐test dates were also required and obtained by computations equivalent to multiple linear regression from residuals for MY, FP and PP at these test‐dates:

$${\hat{e}}_{\mathrm{BCS}}^{*}=\left[R_{\mathrm{BCS}-\mathrm{MY},\;\mathrm{FP},\mathrm{PP}}⊗I^{*}\right]{\left[R_{\mathrm{MY},\;\mathrm{FP},\mathrm{PP}}⊗I^{*}\right]}^{-1}\left[\begin{array}{c} {\hat{e}}_{\mathrm{MY}}\\ {\hat{e}}_{\mathrm{FP}}\\ {\hat{e}}_{\mathrm{PP}} \end{array}\right]$$

where $R_{\mathrm{BCS}-\mathrm{MY},\mathrm{FP},\mathrm{PP}}$ represent the residual covariances between BCS and MY, FP and PP. The matrix $R_{\mathrm{MY},\mathrm{FP},\mathrm{PP}}$ represent the covariances among MY, FP and PP.

Then, the prdBCS values were screened to keep those aligned with observed values that were not used in the variance component estimation (**obsBCS**). Pearson correlation between the prdBCS and obsBCS, prediction error (PE: prdBCS – obsBCS), absolute prediction error (APE: |prdBCS – obsBCS|) and root mean squared error (RMSE) were calculated to evaluate the goodness of the predictions. The agreement between the prdBCS and obsBCS was analysed with Cohen's kappa coefficient. Cohen's kappa coefficient can result in values between −1 and +1, where 0 represents the amount of agreement that can be expected from random chance and 1 represents perfect agreement. While kappa values below 0 are possible, Cohen notes they are unlikely in practice (McHugh, 2012). The results of the kappa test can be interpreted according to the classification *k* < 0.00 = poor, 0.00–0.20 = slight, 0.21–0.40 = fair, 0.41–0.60 = moderate, 0.61–0.80 = substantial and 0.81–1.00 = almost perfect agreement (Landis & Koch, 1977).

## 3 RESULTS

**FIGURE 1:**
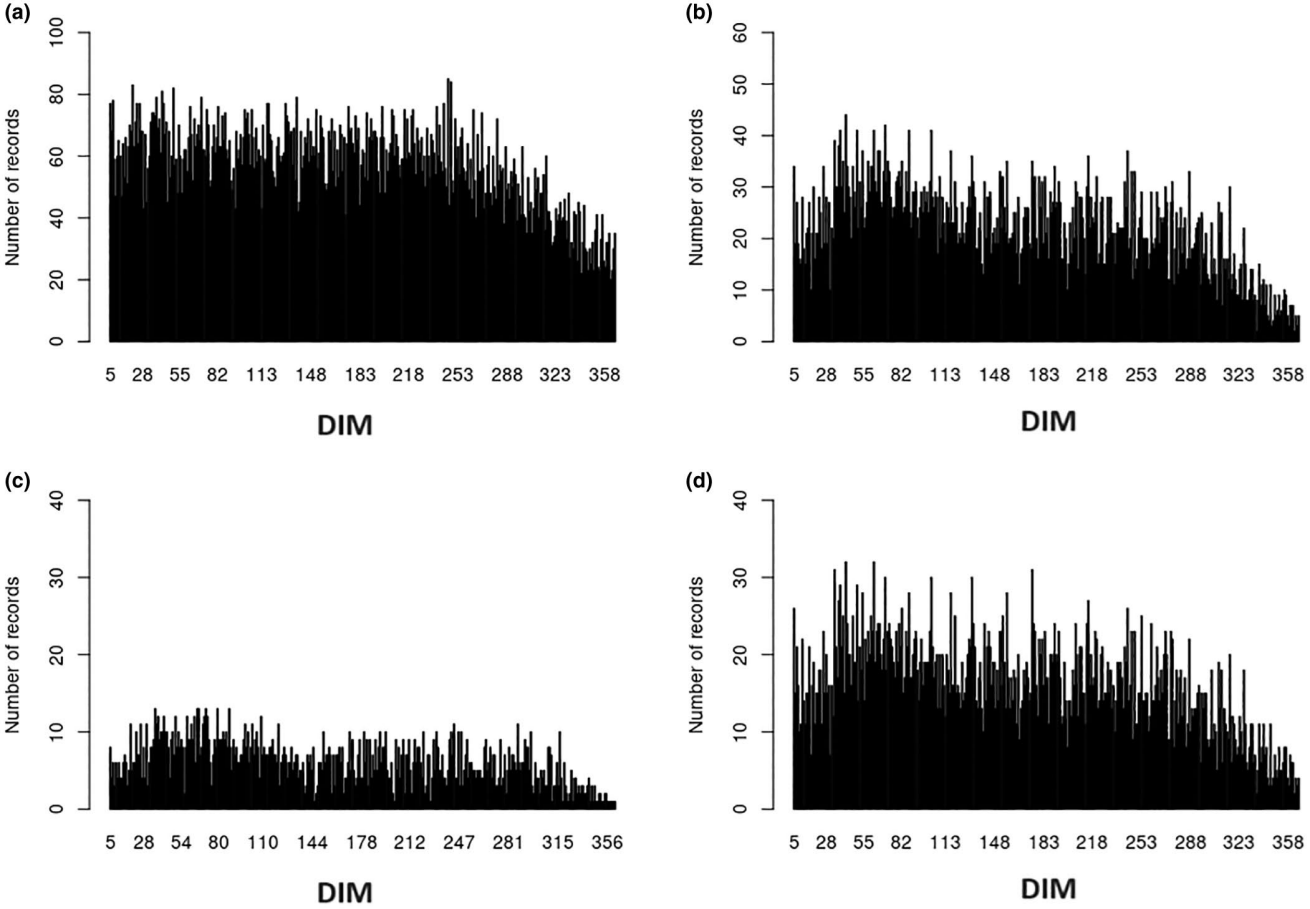
Number of records according to days in milk (DIM) for milk yield traits (a), body condition score (BCS) in the full dataset (b), BCS in the calibration dataset (c) and BCS in the validation dataset (d).

The descriptive statistics for the studied traits are presented in Table 1. Daily milk yield averaged 23.0 kg (4.00% fat and 3.36% protein) and the mean BCS was 4.97 BCS units. Number of records according to DIM for milk traits (MY, FP and PP) and BCS are presented in Figure 1. The quantity of BCS records reduced from the start to the end of the lactation. The number of records ranged from 20 to 85 (mean = 57.3) per DIM for milk yield traits, 2–44 (mean = 22.1) for BCS records in the full dataset, 1–13 (mean = 6.2 records) for BCS records used for estimation of solutions and 2–32 (mean = 16.1 records) for BCS records used for the validation of the prediction. Genetic parameters estimated for the examined traits are presented in Table 2. Average daily heritability for BCS was 0.11 and its average daily genetic correlations with MY, FP and PP were −0.25, −0.07 and 0.09, respectively.

**TABLE 1:** Descriptive statistics for milk yield, milk composition and body condition score (BCS) data used.

	Records (animals)	Mean (SD)	Mean (SD) days in milk (DIM)
MY (kg)	20,698 (2166)	23.00 (6.10)	170 (98.5)
FP (%)	20,698 (2166)	4.00 (0.65)	170 (98.5)
PP (%)	20,698 (2166)	3.36 (0.35)	170 (98.5)
BCS^a^	7985 (2166)	4.97 (1.01)	160 (95.0)
BCS^b^	2166 (2166)	4.95 (1.07)	155 (94.2)
BCS^c^	5819 (2166)	4.98 (0.99)	162 (95.2)

Abbreviations: FP, fat percentage; MY, milk yield; PP, protein percentage.

^a^ BCS records in the full dataset.

^b^ BCS records in the calibration dataset.

^c^ BCS records in the validation dataset.

**TABLE 2:** Average (SD) daily genetic correlations (above the diagonal), daily heritabilities (diagonal) and permanent environmental correlations (below the diagonal) of milk yield, fat percentage, protein percentage and body conduction score (BCS).

	MY	FP	PP	BCS
MY (kg)	0.16 (0.02)	−0.44 (0.12)	−0.45 (0.05)	−0.25 (0.23)
FP (%)	−0.38 (0.10)	0.22 (0.04)	0.61 (0.08)	−0.07 (0.09)
PP (%)	−0.46 (0.13)	0.56 (0.26)	0.25 (0.05)	0.09 (0.14)
BCS^a^	−0.03 (0.30)	0.17 (0.09)	0.16 (0.07)	0.11 (0.03)

Abbreviations: FP, fat percentage; MY, milk yield; PP, protein percentage.

^a^ BCS records in the calibration dataset.

**FIGURE 2:**
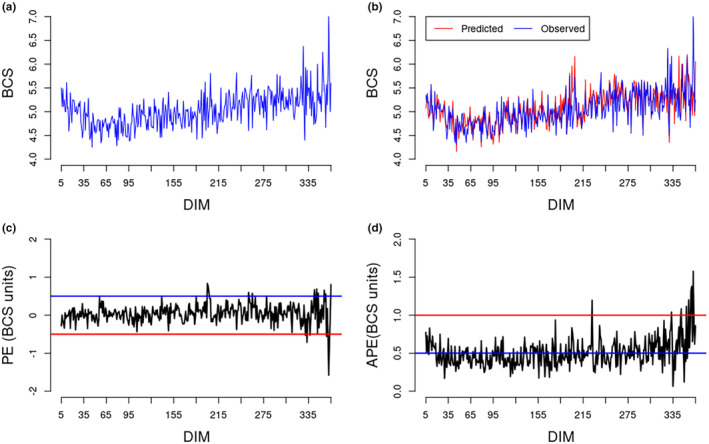
Observed body condition score (BCS) averaged by days in milk (DIM) in the full dataset (a); predicted and observed BCS averaged by DIM in the validation dataset (b); distribution of BCS prediction error averaged according to DIM in the validation dataset (c); distribution of absolute prediction error BCS averaged according to DIM in the validation dataset (d). [Colour figure can be viewed at wileyonlinelibrary.com]

Trend BCS throughout lactation showed the usual decrease in BCS after calving until a few weeks in lactation after which BCS increased again (Figure 2a). Trends observed and predicted BCS throughout lactation followed similar patterns (Figure 2b). Figure 2c shows the mean PE according to DIM. Mean (SD) PE was 0.04 (0.52) and varied from −1.58 to 0.83 BCS units depending on the DIM. Figure 2d shows the average APE according to DIM. Mean (SD) APE was 0.48 (0.53) BCS units. The APE for 3582 (62%) and 4873 (84%) of BCS records was less than 0.50 and 1.00 BCS units, respectively. The APE for only 100 BCS records (less than 2% of all BCS records) was more than 2.00 BCS units. The mean APE was highest for prdBCS of 9 (three records; mean APE = 2.79 BCS units) followed by prdBCS of 8 (55 records; mean APE = 1.19 BCS units), and prdBCS of 2 (16 records; mean APE = 0.90 BCS units).

Distribution of prdBCS against their corresponding obsBCS is presented in Table 3. The results showed that the number of predicted BCS records of classes 5 and 6 is slightly more than those for observed BCS. The correlation between the obsBCS and prdBCS was 0.71 (95% CI = 0.69–0.72). The RMSE of the procedure used in this study was 0.72 BCS units. The kappa test revealed an agreement of κ = 0.67 (95% CI = 0.65–0.69) revealing a substantial agreement between the prdBCS and obsBCS. For 3582 records (61.56% of 5819 BCS records), the prdBCS and obsBCS were identical, while for 1917 records (33% of 5819 BCS records), the prdBCS were deviated from the obsBCS by ±1 BCS units.

**TABLE 3:** The confusion matrix of predicted body condition score (BCS) against their corresponding observed BCS for the validation dataset^a^.

Observed/Predicted	1	2	3	4	5	6	7	8	9	Total (observed)
1	0	0	0	0	0	0	0	0	0	0 (0.00)
2	0	10	1	3	2	0	0	0	0	16 (0.27)
3	0	7	187	117	48	5	0	0	0	364 (6.25)
4	0	3	49	684	412	65	8	0	0	1221 (20.98)
5	0	1	20	305	1912	456	35	2	0	2731 (46.93)
6	1	0	4	49	353	653	76	3	0	1139 (19.58)
7	0	0	1	2	42	121	119	5	0	290 (4.98)
8	0	0	0	1	1	22	14	17	0	55 (0.95)
9	0	0	0	0	2	0	0	1	0	3 (0.05)
Total (predicted)	1 (0.02)	21 (0.36)	262 (4.50)	1161 (19.95)	2772 (47.36)	1322 (22.72)	252 (4.33)	28 (0.48)	0 (0.00)	5819 (1.00)

^a^ The values in brackets are percentages.

## 4 DISCUSSION

Although there are digital‐based procedures to be used in routine body condition scoring (Martins et al., 2020), these procedures need an initial capital cost as well as routine maintenance costs. Therefore, animal scientists and producers are looking for a solution which can predict accurate BCS data routinely at little to no marginal cost. Using mid‐infrared (MIR) spectra of the milk has been suggested as a potential solution to predict BCS in dairy cows; however, accurate alignment of BCS data and MIR spectra is needed for calibration process in this procedure. De Vries et al. (2013) developed regression equations based on milk yield, milk composition (fat, protein and lactose) and body weight to predict BCS. Although recording live weight is not technologically arduous, it requires labour time and thus the number of body weight records per animal is limited. Therefore, using body weight for predicting BCS could not be considered as a solution with a high penetrance rate among dairy cow producers. However, lactating dairy cows are milked daily; thus, using milk yield and composition to predict BCS could be rapidly adopted with a high penetrance rate at minimal marginal cost. In this study, the ability of RR‐TDM to predict BCS for the entire lactation using a single BCS record along with available test‐day records of milk yield and composition was investigated. The results showed that although RR‐TDM tends to slightly overestimate BCS, it has the potential to predict BCS of dairy cows throughout lactation with moderately high accuracy from very limited information. The prediction accuracy was lower for DIM 306–365 than those found for DIM 5–305 which can be attributed, at least in part, to lower number of milk production and BCS records in the final 2 months of lactation. Therefore, using a bigger dataset or excluding data on DIM >305, may result in higher accuracy and higher correlation between observed and predicted BCS. In addition, higher APE was found for BCS class 8 and 9, two most frequent BCS classes in the last 2 months of lactation, which can partly explain why lower accuracy was found for DIM 306–365 than the rest of lactation period. Mayeres et al. (2004) reported that RR‐TDM has the potential to predict milk yield and composition with high accuracy for most of the lactation period, but lower accuracy for DIM 306–365. The RMSE of the procedure used in this study was 0.72 BCS units (equal to 0.36 BCS units in the 5‐point scoring system). De Vries et al. (2013) used milk yields, milk composition (fat, protein and lactose) and body weight to predict BCS (5‐point scoring system) throughout lactation for Holstein cows and reported a RMSE of 0.31 BCS units. Even with the best model possible, many predictions will still differ from the observed values; the results of this study demonstrate that the RR‐TDM can use records of current BCS and milk traits to predict BCS change throughout lactation with relatively high accuracy. This method could be easily incorporated into recording systems for predicting BCS for all DIM for which milk yield or composition data are available. Thus, compiled data on BCS could also be used in genetic evaluations either as a goal trait itself or as a predictor of another trait of importance (e.g. health and fertility). In addition, the procedure introduced by this study can use records of current BCS and milk traits to predict future BCS and could be considered an important tool for herd management.

## 5 CONCLUSIONS

This study showed that RR‐TDM has the potential to predict BCS of dairy cows throughout lactation with moderately high accuracy from very limited, but routinely available Dairy Herd Information (DHI). This method can predict BCS for all DIM for which milk yield or composition data are available. Several conclusions and implications can be drawn here. First milk yield, fat and protein percentages are only the minimum information available through DHI. We can hypothesize that using MIR spectra‐based predictions can be a better proxy than fat and protein percentages and should improve BCS predictions. Not only level of BCS at a given DIM, but also changes of BCS as modelled have the potential to allow inference on status of the cows given their feeding, health or welfare. Research is needed to validate if regular computation and monitoring of predicted BCS compared to observed (i.e. lower than expected BCS) can also help identify cows that are more vulnerable to welfare issues as their susceptibility to heat stress. Moreover, research should show if predicted BCS, especially when predicted using external information (e.g. MIR), can also be used as an additional trait in current genetic evaluation systems for BCS, where direct observed BCS recording is limited to type appraisal systems and therefore mostly only done once in first lactation. Finally, BCS is often used as a predictor of other traits of importance like fertility. Again, the potential usefulness of predicted BCS in this context will need additional research.

## AUTHOR CONTRIBUTIONS

Hadi Atashi: Conceptualization, formal analysis, investigation, methodology and writing the original draft; Yansen Chen, José Chelotti and Pauline Lemal: Reviewing and editing the draft; Nicolas Gengler: Conceptualization, methodology, funding acquisition, project administration, resources, supervision and reviewing and editing the draft. All the authors made their contributions to later versions. All the authors read and approved the final manuscript.

## CONFLICT OF INTEREST STATEMENT

The authors of this work declare that there are no conflicts of interest.

## Data Availability

The data analysed during this study are available through the corresponding author upon reasonable request.

## References

[jbg12890-bib-0001] Bastin, C. , Laloux, L. , Gillon, A. , Bertozzi, C. , Vanderick, S. , & Gengler, N. (2007). First results of body condition score modeling for Walloon Holstein cows. Interbull Bulletin, 37, 170–174.

[jbg12890-bib-0002] Berry, D. , Lee, J. , Macdonald, K. , & Roche, J. (2007). Body condition score and body weight effects on dystocia and stillbirths and consequent effects on postcalving performance. Journal of Dairy Science, 90(9), 4201–4211. 10.3168/jds.2007-0023 17699038

[jbg12890-bib-0003] Berry, D. P. , Buckley, F. , Dillon, P. , Evans, R. , Rath, M. , & Veerkamp, R. (2003). Genetic relationships among body condition score, body weight, milk yield, and fertility in dairy cows. Journal of Dairy Science, 86(6), 2193–2204. 10.3168/jds.S0022-0302(03)73809-0 12836956

[jbg12890-bib-0004] Bewley, J. , & Schutz, M. (2008). An interdisciplinary review of body condition scoring for dairy cattle. The Professional Animal Scientist, 24(6), 507–529. 10.15232/S1080-7446(15)30901-3

[jbg12890-bib-0005] De Vries, A. , Barbosa, L. , Du, F. , Gay, K. , Kaniyamattam, K. , & Maltz, E. (2013). *Prediction of body condition scores in dairy cattle from daily measurements of body weights and milk composition*. Paper presented at the 6th European Conference on Precision Livestock Farming, Leuven; Belgium.

[jbg12890-bib-0006] Dechow, C. , Rogers, G. , Sander‐Nielsen, U. , Klei, L. , Lawlor, T. , Clay, J. , Freeman, A. , Abdel‐Azim, G. , Kuck, A. , & Schnell, S. (2004). Correlations among body condition scores from various sources, dairy form, and cow health from the United States and Denmark. Journal of Dairy Science, 87(10), 3526–3533. 10.3168/jds.S0022-0302(04)73489-X 15377632

[jbg12890-bib-0007] Earle, D. F. (1976). A guide to scoring dairy cow condition. Journal of Agriculture, Victoria, 74, 228–231.

[jbg12890-bib-0008] Edmonson, A. , Lean, I. , Weaver, L. , Farver, T. , & Webster, G. (1989). A body condition scoring chart for Holstein dairy cows. Journal of Dairy Science, 72(1), 68–78. 10.3168/jds.S0022-0302(89)79081-0

[jbg12890-bib-0009] Frizzarin, M. , Gormley, I. , Berry, D. , & McParland, S. (2023). Estimation of body condition score change in dairy cows in a seasonal calving pasture‐based system using routinely available milk mid‐infrared spectra and machine learning techniques. Journal of Dairy Science, 106(6), 4232–4244. 10.3168/jds.2022-22394 37105880

[jbg12890-bib-0010] Garnsworthy, P. C. (2006). Body condition score in dairy cows: Targets for production and fertility. Recent Advances in Animal Nutrition, 2006, 61–86.

[jbg12890-bib-0011] Landis, J. R. , & Koch, G. G. (1977). The measurement of observer agreement for categorical data. Biometrics, 33, 159–174. 10.2307/2529310 843571

[jbg12890-bib-0012] Macdonald, K. , & Roche, J. (2004). Condition scoring made easy. In Condition scoring dairy herds (1st ed.). Dexcel Ltd.

[jbg12890-bib-0013] Martins, B. , Mendes, A. , Silva, L. , Moreira, T. , Costa, J. , Rotta, P. , Chizzotti, M. L. , & Marcondes, M. (2020). Estimating body weight, body condition score, and type traits in dairy cows using three dimensional cameras and manual body measurements. Livestock Science, 236, 104054. 10.1016/j.livsci.2020.104054

[jbg12890-bib-0014] Mayeres, P. , Stoll, J. , Bormann, J. , Reents, R. , & Gengler, N. (2004). Prediction of daily milk, fat, and protein production by a random regression test‐day model. Journal of Dairy Science, 87(6), 1925–1933. 10.3168/jds.S0022-0302(04)73351-2 15453510

[jbg12890-bib-0015] Mayeres, P. , Stoll, J. , Reents, R. , & Gengler, N. (2002). Alternative modeling of fixed effects in test day models to increase their usefulness for management decisions. Interbull Bulletin, 29, 128.

[jbg12890-bib-0016] McHugh, M. L. (2012). Interrater reliability: The kappa statistic. Biochemia Medica, 22(3), 276–282. 23092060 PMC3900052

[jbg12890-bib-0017] Mee, J. , & Boyle, L. (2020). Assessing whether dairy cow welfare is “better” in pasture‐based than in confinement‐based management systems. New Zealand Veterinary Journal, 68(3), 168–177. 10.1080/00480169.2020.1721034 31973680

[jbg12890-bib-0018] Misztal, I. , Tsuruta, S. , Lourenco, D. , Masuda, Y. , Aguilar, I. , Legarra, A. , & Vitezica, Z. (2018). Manual for BLUPF90 family of programs. University of Georgia.

[jbg12890-bib-0019] Mulvany, P. (1977). Dairy cow condition scoring. Handout No. 4468 (Vol. 4, pp. 349–353). National Institute for Research in Dairying.

[jbg12890-bib-0020] Ptak, E. , & Schaeffer, L. (1993). Use of test day yields for genetic evaluation of dairy sires and cows. Livestock Production Science, 34(1–2), 23–34. 10.1016/0301-6226(93)90033-E

[jbg12890-bib-0021] Roche, J. , Lee, J. , Macdonald, K. , & Berry, D. (2007). Relationships among body condition score, body weight, and milk production variables in pasture‐based dairy cows. Journal of Dairy Science, 90(8), 3802–3815. 10.3168/jds.2006-740 17638991

[jbg12890-bib-0022] Roche, J. R. , Friggens, N. C. , Kay, J. K. , Fisher, M. W. , Stafford, K. J. , & Berry, D. P. (2009). Invited review: Body condition score and its association with dairy cow productivity, health, and welfare. Journal of Dairy Science, 92(12), 5769–5801. 10.3168/jds.2009-2431 19923585

[jbg12890-bib-0023] Roche, J. R. , Kay, J. K. , Friggens, N. C. , Loor, J. J. , & Berry, D. P. (2013). Assessing and managing body condition score for the prevention of metabolic disease in dairy cows. Veterinary Clinics: Food Animal Practice, 29(2), 323–336. 10.1016/j.cvfa.2013.03.003 23809894

[jbg12890-bib-0024] Souissi, W. , & Bouraoui, R. (2019). Relationship between body condition score, milk yield, reproduction, and biochemical parameters in dairy cows. In N. M'Hamdi (Ed.), Lactation in farm animals‐biology, physiological basis, nutritional requirements, and Modelization. Intechopen. 10.5772/intechopen.85343

[jbg12890-bib-0025] Swalve, H. (1995). The effect of test day models on the estimation of genetic parameters and breeding values for dairy yield traits. Journal of Dairy Science, 78(4), 929–938. 10.3168/jds.S0022-0302(95)76708-X 7790586

[jbg12890-bib-0026] Van Vleck, L. D. (1987). Contemporary groups for genetic evaluations. Journal of Dairy Science, 70(11), 2456–2464. 10.3168/jds.S0022-0302(87)80309-0 3693648

[jbg12890-bib-0027] Waltner, S. , McNamara, J. , & Hillers, J. (1993). Relationships of body condition score to production variables in high producing Holstein dairy cattle. Journal of Dairy Science, 76(11), 3410–3419. 10.3168/jds.S0022-0302(93)77679-1 8270683

[jbg12890-bib-0028] Wildman, E. , Jones, G. , Wagner, P. , Boman, R. , Troutt, H., Jr. , & Lesch, T. (1982). A dairy cow body condition scoring system and its relationship to selected production characteristics. Journal of Dairy Science, 65(3), 495–501. 10.3168/jds.S0022-0302(82)82223-6

[jbg12890-bib-0029] Zink, V. , Stipkova, M. , & Lassen, J. (2011). Genetic parameters for female fertility, locomotion, body condition score, and linear type traits in Czech Holstein cattle. Journal of Dairy Science, 94(10), 5176–5182. 10.3168/jds.2010-3644 21943767

